# Supplementary material for: Complexome profiling of the Chlamydomonas psb28 mutant reveals TEF5 as an early PSII assembly factor
Source: Plant Cell. 2025 Mar 17;37(6):koaf055. doi: 10.1093/plcell/koaf055 (PMC12164590; doi:10.1093/plcell/koaf055)
Supplement: koaf055_Supplementary_Data [file koaf055_supplementary_data.zip › psb28_interactive.zip!/plots/intensity/qualityPlots/peptides/ATPVA1_Cre10.g461050.t1.2.html]

### Cre10.g461050.t1.2

Trivial name: ATPVA1  
SeqCoverage [median]: 46.30  
Mass: 68.39 kDa  
  
 

| Sequence | MissedCleavage | Mass [Da] | wtIntensity | muIntensity | Score |
| --- | --- | --- | --- | --- | --- |
| ALEDEFR | 0 | 878.41 | 1313.51 | 22187.58 | 134.85 |
| AVYEELHDR | 0 | 1130.54 | 0.00 | 2079.77 | 54.98 |
| AVYEELHDRFR | 1 | 1433.71 | 2750.39 | 11727.87 | 52.79 |
| DALAETDKIVLETAR | 1 | 1643.87 | 7356.86 | 22898.93 | 131.40 |
| DMGYHFSMMGDSTSR | 0 | 1720.66 | 0.00 | 3404.48 | 78.50 |
| ESDYGYIR | 0 | 1001.45 | 674.05 | 5742.30 | 104.37 |
| ESDYGYIRK | 1 | 1129.54 | 0.00 | 2079.93 | 66.62 |
| EVLQKEDELNEIVQLVGK | 1 | 2082.12 | 8067.30 | 0.00 | 99.71 |
| FEDPAEGEEVVR | 0 | 1375.63 | 0.00 | 1023.31 | 83.00 |
| FEDPAEGEEVVRR | 1 | 1531.73 | 0.00 | 341.10 | 52.34 |
| FIKDDYLQQNSFTK | 1 | 1745.86 | 8314.19 | 19059.57 | 116.19 |
| ITGGDIYGIVNENSLIEHR | 0 | 2099.06 | 21339.53 | 33205.99 | 134.21 |
| ITLNVIK | 0 | 799.52 | 3856.50 | 23745.01 | 138.85 |
| KVSGPVVVADHMAGAAMYELVR | 1 | 2299.18 | 0.00 | 6713.48 | 89.67 |
| LAEMPADSGYPAYLGAR | 0 | 1780.85 | 4786.96 | 34968.58 | 160.22 |
| LASFYER | 0 | 884.44 | 0.00 | 26846.68 | 121.29 |
| LATAAIEK | 0 | 815.48 | 15659.07 | 42352.34 | 163.90 |
| LKAVYEELHDRFR | 2 | 1674.88 | 0.00 | 1165.95 | 29.69 |
| LLADTPLLTGQR | 0 | 1296.74 | 815.02 | 48736.86 | 176.60 |
| LTSQKFEDPAEGEEVVR | 1 | 1932.94 | 12170.13 | 24196.56 | 163.41 |
| LTSQKFEDPAEGEEVVRR | 2 | 2089.04 | 0.00 | 2506.71 | 56.96 |
| NISTFHR | 0 | 873.45 | 1704.72 | 9214.44 | 113.70 |
| QIAIDSGDVFIPR | 0 | 1429.76 | 2785.85 | 0.00 | 94.31 |
| SLEPFYDKFDPEFVQLR | 1 | 2129.05 | 7052.85 | 0.00 | 114.59 |
| SVEMMR | 0 | 751.34 | 583.64 | 8508.28 | 80.38 |
| TVISQALSK | 0 | 945.55 | 7302.47 | 28990.41 | 131.22 |
| VGTDNLIGEIIR | 0 | 1298.72 | 6062.74 | 4496.20 | 124.67 |
| VSGPVVVADHMAGAAMYELVR | 0 | 2171.09 | 2081.62 | 18394.62 | 96.55 |
| WAEALREISGR | 1 | 1286.67 | 0.00 | 606.04 | 38.63 |
|  |  |  | 114677.4 | 405193.0 |  |
